# Increased expression of collagens, transforming growth factor-β1, and -β3 in gluteal muscle contracture

**DOI:** 10.1186/1471-2474-11-15

**Published:** 2010-01-25

**Authors:** Chen-Guang Zhao, Xi-Jing He, Bin Lu, Hao-Peng Li, An-Jing Kang

**Affiliations:** 1 Orthopedic center of Chinese PLA, Urumqi General Hospital of Lanzhou Military Region, Urumqi, Xinjiang, PR China; 2 Department of Orthopedic Surgery, The 2nd Affiliated Hospital of Medical College, Xi'an JiaoTong University, Xi'an, Shaanxi, PR China; 3 Department of Orthopedic Surgery, Chinese People's Armed Police Troops Shaanxi Province Corps Hospital, Xi'an, Shaanxi, PR China; 4 Department of Pathology, The 2nd Affiliated Hospital of Medical College, Xi'an JiaoTong University, Xi'an, Shaanxi, PR China

## Abstract

**Backgroud:**

Gluteal muscle contracture (GMC) is a multi-factor human chronic fibrotic disease of the gluteal muscle. Fibrotic tissue is characterized by excessive accumulation of collagen in the muscle's extracellular matrix. Transforming growth factor (TGF)-β1 and -β2 are thought to play an important role in fibrogenesis, while TGF-β3 is believed to have an anti-fibrotic function. We hypothesize that the expression of collagen and TGF-βs would be up-regulated in GMC patients.

**Methods:**

The expression of collagen type I, type III and TGF-βs were studied in 23 fibrotic samples and 23 normal/control samples in GMC patients using immunohistochemistry, reverse transcription and polymerase chain reaction (RT-PCR) and western bolt analysis.

**Results:**

Compared to the unaffected adjacent muscle, increased expression of TGF-β1 and -β3 was associated with deposition of collagen type I and type III in the fibrotic muscle of the GMC patients at the mRNA level. Strong up-regulation of these proteins in fibrotic muscle was confirmed by immunohistochemical staining and western blot analysis. TGF-β2 was not up-regulated in relation to GMC.

**Conclusion:**

This study confirmed our hypothesis that collagen types I, III, TGF-β1 and TGF-β3 were up-regulated in biopsy specimens obtained from patients with GMC. Complex interaction of TGF-β1 with profibrotic function and TGF-β3 with antifibrotic function may increase synthesis of collagens and thereby significantly contribute to the process of gluteal muscle scarring in patients with GMC.

## Background

Gluteal muscle contracture (GMC) is a clinical syndrome with multiple etiologies, either congenital or acquired, GMC is not uncommon and exists worldwide [1-5]. Clinically, GMC progresses through distinct stages. The earliest stage is characterized by unnoticed difficulties in adducting the hip or squatting, and over time, it gives rise to serious functional disorders of the hip. Although abnormal scarring is a known phenomenon in the fibrotic muscle (contraction band) of GMC patients, the mechanism of this human chronic fibrotic muscle disorder is not known.

Transforming growth factor (TGF)-βs are members of a superfamily of polypeptides which play a key role in cell regulation, angiogenesis, embryo development, apoptosis induction, and wound healing [6-8]. Three isoforms of TGF-β are known to exist in mammals, namely TGF-β1, -β2, and -β3, each with distinct roles in wound repair. TGF-β1 and -β2 are potent profibrotic factors thought to be involved in the pathogenesis of many fibrotic diseases and are important in almost every step in the process of tissue fibrosis by simultaneously signaling fibroblasts to increase the synthesis of matrix proteins, decrease the production of matrix-degrading proteases, and increase the production of inhibitors of these proteases [9,10]. Both *in vitro *and *in vivo *studies have revealed an over-expression of TGF-β1 and TGF-β2 in fibrotic lesions, and convincingly demonstrated that blocking the bioactivity of TGF-β1 and -β2 could suppress collagen production and subsequently could modulate the fibrotic process [11,12]. In contrast, TGF-β3 reduces the fibrotic response. The exogenous addition of TGF-β3 peptide has an anti-scarring properties as well [13,14].

Although fibrogenic roles for each of the three isoforms of TGF-βs have been documented during fibrosis in various tissues and organs, their roles in GMC patients are not known. This study was designed, therefore, with an expectation of finding an increased expression of collagen types I and III, and TGF-βs in this fibrotic disease of gluteal muscle.

## Methods

### Tissue specimens and antibodies

Between January 2007 and March 2008, 46 samples (23 contraction bands and 23 adjacent normal muscle samples) of GMC patients were enrolled in this study in the Department of Orthopaedic Surgery of The 2nd Affiliated Hospital of Medical College of Xi'an JiaoTong University. All our studies were permitted by the Ethical Board of The 2nd Affiliated Hospital of Medical College of Xi'an JiaoTong University and followed the Declaration of Helsinki Guidelines. Informed consent was obtained from all of the patients. All 23 patients (10 males and 13 females between the ages of 7 to 27 years, 8 years of course at least) were carried out with contraction band releasing surgery as we described before [1]. Tissues used for RT-PCR and western blot analysis were immediately frozen in liquid nitrogen and stocked at -80°C. Mouse anti-human monoclonal antibodies against collagen types I and III were purchased from Abcam (Cambridge, UK), and mouse anti-human monoclonal antibodies against TGF-β1, -β2, and β-3 were purchased from Santa Cruz Biotechnology, Inc. (Santa Cruz, CA, US). Second antibody for immunohistochemical analyses (biotinylated goat anti-mouse IgG antibody) was obtained from Vector Laboratories (Burlingame, CA, USA).

### Histology and immunohistochemistry

Tissue samples were fixed in 4% paraformaldehyde at 4°C for 24 hours. Paraffin sections (4 um) for histologic analysis were stained with hematoxylin/eosin, Masson trichrome, and picrosirius. For immunohistochemical analysis, paraffin sections were deparaffinized with xylene, rinsed thoroughly with ethanol, then soaked in 0.3% hydrogen peroxide in methanol to inactivate endogenous peroxidase activity. After mild treatment with 0.05% trypsin-EDTA, the sections were incubated with 10% goat serum to block non-specific protein binding. The blocked sections were incubated with antibodies against collagen types I and III (1:800), or antibodies against TGF-β1, -β2, and -β3 (1:200) at 4°C overnight. After incubation with the primary antibody, the sections were incubated with second antibody and alkaline phosphatase streptavidin (Vector Laboratories, Burlingame, CA, USA). The sections were stained using a Vector Red Alkaline Phosphatase Substrate Kit (Vector Laboratories) according to the manufacturer's instructions and counterstained with Mayer's hematoxylin. One section from each series was incubated with PBS as the first antibody as a negative control. The staining intensities of collagen types I and III, TGF-β1, -β2, and -β3 in the contraction band and unaffected adjacent muscle were graded semi-quantitatively using the following scale: (-) = no staining; (+) = weak staining; (++) = moderate staining; and (+++) = strong staining.

### Reverse transcription and polymerase chain reaction (RT-PCR)

Total RNA was extracted from samples using Trizol reagent (Invitrogen Corp., Carlsbad, CA, USA) according to the manufacturer's protocol. Five micrograms of total RNA template were used to make cDNA by using a RevertAid First Strand cDNA Synthesis Kit (Fermentas, Vilnius, Lithuania), using random hexamers as the first-strand primer. A polymerase chain reaction (PCR) was performed using TaKaRa Ex Taq HS (TaKaRa, Da Lian, China) and the synthetic gene-specific primers for collagen types I and III, TGF-β1, -β2, -β3, and β-actin used in the RT-PCR are listed in Table 1. All target sequences were separately amplified for 35 cycles. Samples of each reaction product were separated by agarose gel electrophoresis, visualized by ethidium bromide staining, and photographed with 290-nm ultraviolet illumination. The density of each band was measured by densitometry. Relative expression of collagen types I III, TGF-β1, -β2, and -β3 mRNA were normalized to the expression of the internal control (β-actin).

**Table 1:** Synthetic gene-specific primer sequences for collagen types I and III and for TGF-β1, -β2, -β3 and β-actin

Gene		Primers	Product Size
Collagen types I	Forward	GTCGAGGGCCAAGACGAAG	143 bp
	Reverse	CAGATCACGTCATCGCACAAC	
Collagen type III	Forward	TGGTCCCCAAGGTGTCAAAG	117 bp
	Reverse	GGGGGTCCTGGGTTACCATTA	
TGF-β1	Forward	GGCCAGATCCTGTCCAAGC	201 bp
	Reverse	GTGGGTTTCCACCATTAGCAC	
TGF-β2	Forward	TGCCATCCCGCCCACTTTC	252 bp
	Reverse	GAGAGCCATTCGCCTTCTGC	
TGF-β3	Forward	CTGTGCGTGAGTGGCTGTTG	406 bp
	Reverse	GTGCTGTGGGTTGTGTCTGC	
β-actin	Forward	ATCGTGCGTGACATTAAGGAGAAG	179 bp
	Reverse	AGGAAGGAAGGCTGGAAGAGTG	

### Western blot analysis

Tissue protein extracts were prepared using a modified RIPA buffer (50 mM Tris-HCl [pH 7.4], 1% NP-40, 150 mM NaCl, and 1 mM EDTA) supplemented with a cocktail of protease inhibitors (1 mM PMSF [1 μg/mL of aprotinin, leupeptin, and pepstatin]). Tissue was homogenized at 4°C and the resulting homogenate centrifuged for 12,000 rpm for 15 min at 4°C to remove cell debris. Sample protein concentrations were quantified using the BCA protein assay (Pierce, Rockford, IL, USA). Equivalent levels of protein (20 μg) were analyzed by SDS-PAGE and subsequently transferred to polyvinylidene difuoride (PVDF) membranes (Bio-Rad, Hercules, CA, USA). The membranes were than blocked with 10% fat-free skim milk powder in phosphate-buffered saline containing 0.1% Tween 20, and incubated with antibodies against TGF-β1, -β2, and TGF-β3 (1:400). After a series of brief PBST washes, the membranes were then incubated with the appropriate species-specific horseradish peroxidase-conjugated antibodies (1:5000; Jackson ImmunoResearch, West Grove, PA, USA). Antibody-specific bands were visualized using standard ECL chemiluminescence reagents (Pierce) and photographed. The density of each band was measured by densitometry. The relative expression of collagen types I and III, TGF-β1, -β2, and-β3 protein were normalized to the expression of the internal control (β-actin).

### Statistical analyses

Densitometric measurement was performed using a GS-700 Imaging Densitometer with Multi-Analyst software (Bio-Rad). All values were expressed as the means ± SD. The paired T-test was used to examine differences between paired samples. Statistical analysis was performed using SPSS 13.0 software and *P *values < 0.05 were considered significant.

## Results

### Histology analysis

**Figure 1:**
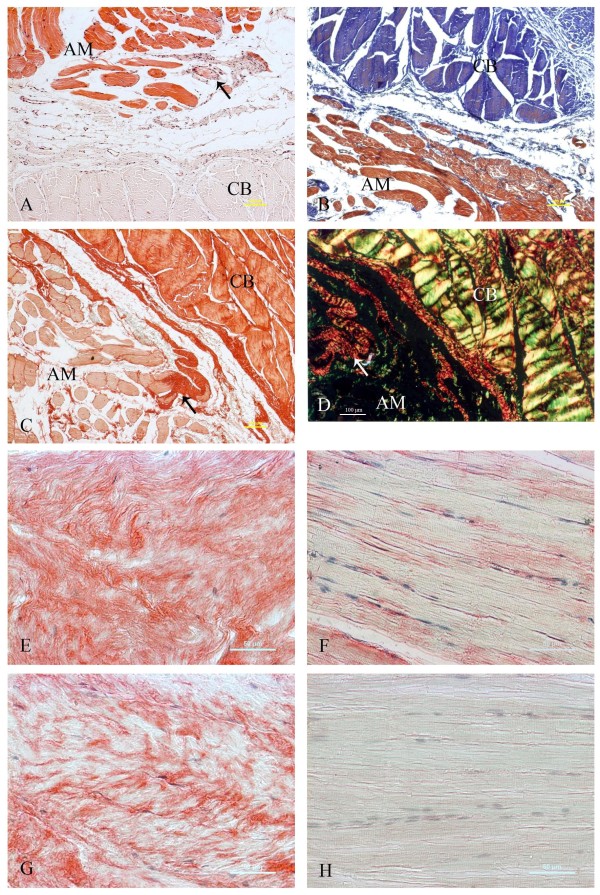
**Histochemical analyses for muscle lesion and immunohistochemical analyses for collagen type I and type III**. (A) Hematoxylin-eosin, (B) Masson trichrome and (C) picrosirius staining of the boundary between the contraction band (CB) and unaffected adjacent muscle (AM). Arrows indicate muscles that are undergoing fibrosis. (D) picrosirius staining of the boundary viewed under polarized light microscopy (collagen type I is yellow and type III is green). (E, F, G, H) Immunohistochemical staining of contraction band (E, G) or unaffected adjacent muscle (F, H) was carried out with antibodies against collagen type I (E, F) or collagen type III (G, H). Proteins specifically recognized by the antibodies are stained red, whereas nuclei are counter-stained blue. Scale bars: 100 μm (A, B, C, D), 50 μm (E, F, G, H).

Histologic examination was performed on sections stained with hematoxylin/eosin (Fig. 1A), Masson trichrome (Fig. 1B), or picrosirius (Fig. 1c). We observed typical fibrotic changes in the contraction band. Irregular broad segments of collagen fibers accumulated in a whirl-like pattern and replaced the original skeletal muscle fibers. A small amount of collagen was also observed within the muscle bundles. We showed that on sections stained with picrosirius, under polarized microscopy, collagen types I and III co-existed, and the staining of type I was more intense (Fig. 1D).

### Expression of collagen types I and III is up-regulated in the contraction band

**Figure 2:**
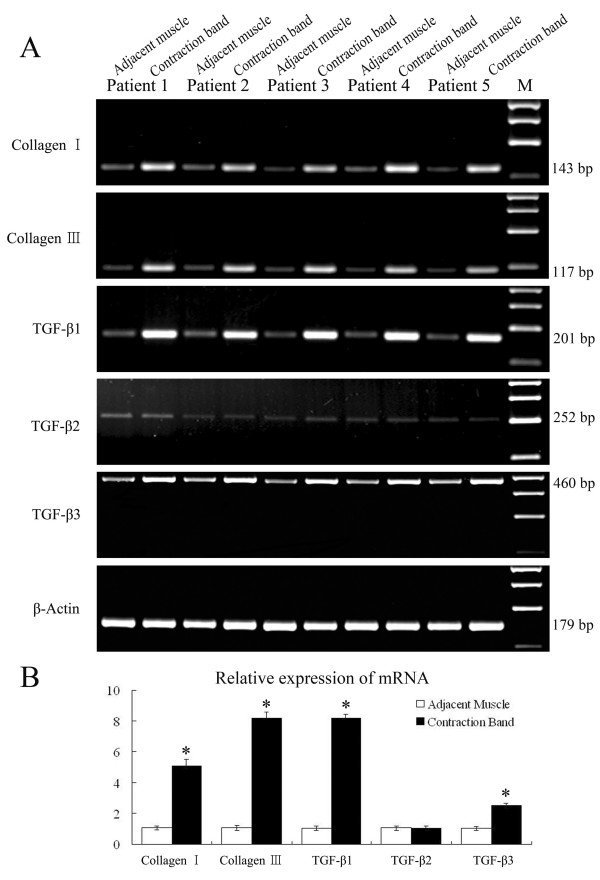
**(A) Expression of collagen types I, type III, TGF-β s and β-actin mRNA in adjacent muscle and contraction band in 5 of the GMC patients by RT-PCR**. (B) Densitometry of mRNA levels of collagen types I, III and TGF-β s in contraction band and adjacent muscle (fold change). The results are shown relative mRNA level of collagen types I, type III, and TGF-β s in contraction band compared with adjacent muscle in 23 GMC patients.

The mRNA levels of collagen types I and III were determined using RT-PCR. Compared with adjacent muscle, the mRNA levels of collagen types I and III were elevated (Fig. 2A; 5 representative cases are shown). Semi-quantitative densitometric analysis of the blots indicated that the levels of collagen types I and III mRNA in the contraction band were increased 5.0- and 8.1-fold, respectively (Fig. 2B; mean of 23 cases; *P *< 0.01), relative to unaffected adjacent muscle in the same patients. To further explore whether the up-regulation of mRNA levels for collagen types I and III leads to increased protein levels, an immunohistochemical analysis was performed to detect collagen types I and III (Fig.1E-1H). Collagen types I and III were weakly present in the adjacent muscle (+). Compared to unaffected adjacent muscle, expression of collagen types I (+++) and III (++) was detected in the contraction band, which is consistent with findings from sections stained with picrosirius (Table 2). All of these indicate an increased expression of collagen types I and III in GMC.

**Table 2:** Semi-Quantitative Scale of Collagen Type I, Type III, TGF-β1, TGF-2 and TGF-β3 in Contraction Band and Adjacent Muscle

	Collagen Type I	Collagen Type III	TGF-β1	TGF-β2	TGF-β3
Adjacent Muscle	+	-			
Fibroblasts			+	**-**	+
Endothelial cell			+	**-**	+
Contraction Band	+++	++			
Fibroblasts			+++	+	++
Endothelial cell			+++	+	++

Semi-quantitative scale: (-) = no staining; (+) = weak staining; (++) = moderate staining; and (+++) = strong staining.

### Expression of TGF-β1 and -β3 mRNA is up-regulated in the contraction band

As TGF-β1, -β2, and -β3 have a close relationship with the process of tissue fibrosis, we first examined the mRNA level in the contraction band by RT-PCR. The expression of TGF-β1 and -β3 was weakly detected in unaffected adjacent muscle, while the expression was increased in the contraction band (Fig. 2A; 5 representative cases are shown). Semi-quantitative densitometric analysis of the blots indicated that the levels of TGF-β1 and -β3 mRNA in the contraction band were increased 8.1 and 2.5-fold, respectively (Fig. 2B; mean of 23 cases; *P *< 0.01), relative to adjacent muscle in the same patients. The expression of TGF-β2 was not increased (*P *> 0.05).

### Immunohistochemical staining for TGF-β1, -β2, and -β3

**Figure 3:**
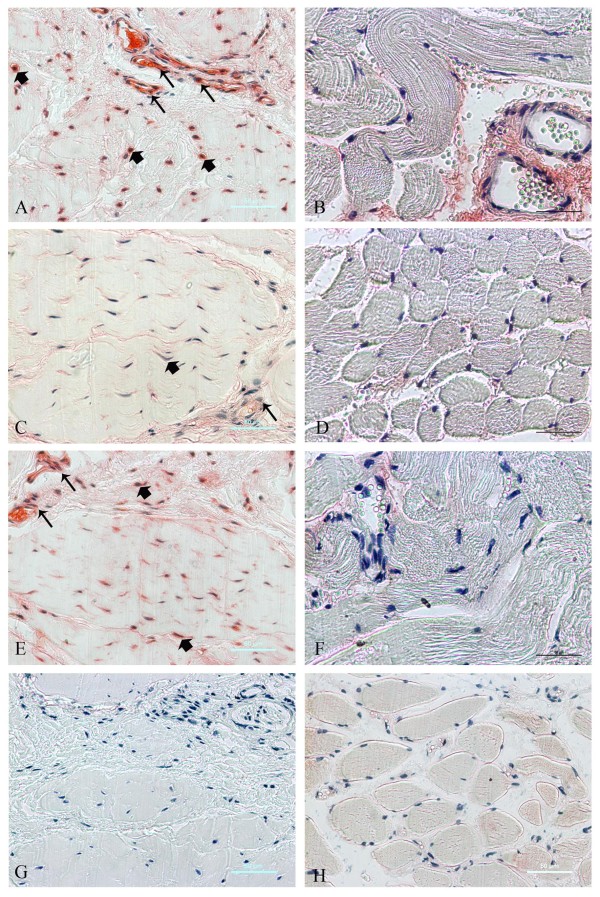
**Immunohistochemical analyses for TGF-β1, TGF-β2 and TGF-β3 in contraction band (A, C, E, G) and unaffected adjacent muscle (B, D, F, H)**. Staining was carried out with antibodies against TGF-β1 (A, B), TGF-β2 (C, D), TGF-β3 (E, F) or without first antibody (control) (G, H). Proteins specifically recognized by the antibodies are stained red, whereas nuclei are counter-stained blue. Positive cells are fibroblasts (arrowheads) and vascular endothelial cell (arrows). Scale bars: 50 μm (A-H).

We then performed immunostaining for TGF-β1, -β2, and TGF-β3 to determine the immunolocalization and level of expression in the contraction band and adjacent muscle. Using immunohistochemical analysis, an increased expression of TGF-β1 and -β3 was detected in the fibroblasts (+++; arrow) and vascular endothelial cells (+++; arrowhead) in the contraction band (Fig. 3A, 3C; Table 2). The expression of TGF-β1 and -3 protein was weakly and sparsely detected in vascular endothelial cells in adjacent muscle sections (+; Fig.3B, 3D; Table 2). The expression patterns of both TGF-β1 and TGF-β3 were similar. Compared to the adjacent muscle (+), no increased cytoplasmic staining for TGF-β2 was detected in the contraction band from the same GMC patient.

### Expression of TGF-β1 and -β3 protein is up-regulated in the contraction band

**Figure 4:**
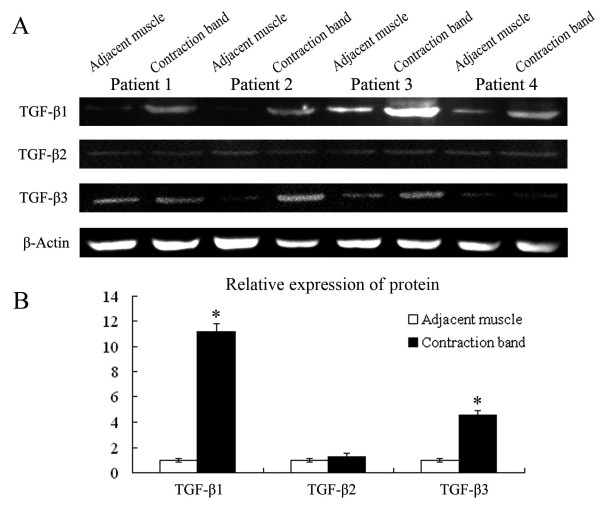
**(A) Expression of TGF-β s and β-actin protein in adjacent muscle and contraction band in 5 of the GMC patients by Western blot analysis**. (B) Densitometry of protein levels of TGF-β s in contraction band and adjacent muscle (fold change). The results are shown relative protein level of TGF-β s in contraction band compared with adjacent muscle in 23 GMC patients.

To further determine the level of protein expression of TGF-β1, -β2, and -β3, we then examined these proteins by western blot analysis. A strongly immunoreactive band using TGF-β1 and -β3 antibodies was detected in all contraction band samples examined; however, the immunoreactive band in adjacent muscle was very weak (Fig.4A; 4 representative cases are shown). The protein expression of TGF-β1 and -β3 in the contraction band were increased 11.2- and 4.5-fold, respectively (Fig. 4B; mean of 23 cases; *P *< 0.01), relative to unaffected adjacent muscle in the same patients. Consistent with the result of mRNA expression, TGF-β2 was not up-regulated (*P *> 0.05).

## Discussion

Although enormous work has been done to investigate the mechanism of many fibrotic diseases, such as keloids, atherosclerosis, liver cirrhosis, lung fibrosis, and kidney fibrosis, the mechanism involved in this fibrotic muscle disease is not known [15-19]. In the current study, we detected the expression and localization of collagen types I and III, and TGF-βs in GMC patients by immunohistochemistry, RT-PCR, and western blot analysis.

Our first experiment demonstrated that collagen types I and III were major components of the contraction band and were remarkably elevated compared to unaffected adjacent muscle. Several reports have documented increased synthesis of extracellular matrix (ECM), including collagen type I, during development of fibrosis in experimental and human diseases. However, in terms of collagen type III, the status of synthesis is controversial. Indeed, some reports have suggested that synthesis is elevated and some have demonstrated that synthesis is unchanged [20]. A possible explanation for this difference could be that cultured fibroblasts obtained from fibrotic lesions have lost specific characteristics. Type III collagen gene expression might be transiently activated in specific stages of development of fibrosis, while those of cultured fibroblasts might have been by chance those inactive in other stages, as supported by the work of Zhang [21].

TGF-βs are pluripotent growth factors and are highly conserved between species. There is 64%-85% amino acid sequence homology between TGF-βs. These TGF-βs isoforms bind to common specific transmembrane receptors (TGF-β receptor types I and II) to target genes via the SMAD family of signal transducing proteins [22]. Previous studies have shown that TGF-βs play pivotal roles in the development of fibrosis in various tissues and organs [15-19]. Their role on transcriptional activation of collagen genes and post-translational modification makes them potential candidates to contribute in matrix remodeling during the fibrosis. Thus, we believe that the formation of GMC is due, at least in part, to an alteration of expression of the TGF-β family. In our study, we found that the mRNA expression of TGF-β1 and TGF-β3 is remarkably increased in contraction band compared with the expression in unaffected adjacent muscles. These observations were enhanced using immunohistochemistry and western blot analysis. From the results of our study, and taking into account the findings of previous studies, it is suggested that up-regulation of TGF-β1 and TGF-β3 in the contraction band in GMC patients contribute to muscle fibrosis. The results also suggest that the process of fibrosis in GMC patients might be a result of competition between the profibrotic function of TGF-β1 and the anti-fibrotic function of TGF-β3. Further study would be done to investigate whether TGF-β1-to-TGF-β3 ratio would influence progresses of fibrosis as some research reported [23].

However, in terms of TGF-β2, there was no evidence indicating a difference between the two kinds of tissues at the two levels. Previous studies have emphasized that TGF-β2 has a potential role in the formation of fibrosis, but the time window is not clear [24]. From our findings in the experiments herein, we hypothesize that TGF-β2 is secreted only at the initial stage and might be an assistant factor for TGF-β1 in fibrosis in gluteal muscle *in vivo*.

Curiously, in the survey of our post-operative patients, most undergo another form of fibrosis (hypertrophic scars in skin or keloids in the incision site) which is in agreement with other reports [25,26]. These patients seemingly have a tendency towards fibrosis in organs. Whether there is a healing-associated gene variation in these patients needs additional work for confirmation.

## Conclusion

The present study confirmed our hypothesis that GMC is a fibrotic disease of gluteal muscle characterized by accumulation of collagen types I and III, and up-regulation of TGF-β1 and -β3. All of these findings could be important in understanding the molecular mechanism of GMC and could provide specific sites for molecular intervention for treatment or arrest of the progression of this disease.

## Competing interests

The authors declare that they have no competing interests.

## Authors' contributions

CGZ and XJH are the lead investigators, and developed the design of the study, carried out data-acquisition, analysis, interpretations, and prepared as primary authors for the manuscript. BL and HPL were responsible for the design, project supervision, and writing of the manuscript. AJK and TL assisted in carrying out data acquisition and were involved in preparing the study design. All authors read, edited, and approved the final manuscript.

## Pre-publication history

The pre-publication history for this paper can be accessed here:

http://www.biomedcentral.com/1471-2474/11/15/prepub
